# Plasma Treated Water Solutions in Cancer Treatments: The Contrasting Role of RNS

**DOI:** 10.3390/antiox10040605

**Published:** 2021-04-14

**Authors:** Eloisa Sardella, Valeria Veronico, Roberto Gristina, Loris Grossi, Savino Cosmai, Marinella Striccoli, Maura Buttiglione, Francesco Fracassi, Pietro Favia

**Affiliations:** 1 CNR- Istituto di Nanotecnologia (CNR-NANOTEC) UoS Bari, c/o Dipartimento di Chimica, Università degli Studi di Bari Aldo Moro, via Orabona, 4, 70126 Bari, Italy; roberto.gristina@cnr.it (R.G.); savino.cosmai@cnr.it (S.C.); francesco.fracassi@uniba.it (F.F.); pietro.favia@uniba.it (P.F.); 2 Dipartimento di Chimica, Università degli Studi di Bari Aldo Moro, via Orabona, 4, 70126 Bari, Italy; valeria.veronico@uniba.it; 3 Dipartimento di “Scienze per la Qualità della Vita” Università di Bologna, Corso d’Augusto, 237, I-47921 Rimini, Italy; loris.grossi@unibo.it; 4 CNR-Istituto per i Processi Chimico-Fisici UoS Bari, via Orabona, 4, 70126 Bari, Italy; m.striccoli@ba.ipcf.cnr.it; 5 Dipartimento di Scienze Biomediche e Oncologia Umana, Scuola di Medicina, Università degli Studi di Bari Aldo Moro, 70124 Bari, Italy; maura.buttiglione@uniba.it

**Keywords:** cold atmospheric plasma, reactive oxygen and nitrogen species, oxidative stress, nitrite, cancer treatment

## Abstract

Plasma Treated Water Solutions (PTWS) recently emerged as a novel tool for the generation of Reactive Oxygen and Nitrogen Species (ROS and RNS) in liquids. The presence of ROS with a strong oxidative power, like hydrogen peroxide (H_2_O_2_), has been proposed as the main effector for the cancer-killing properties of PTWS. A protective role has been postulated for RNS, with nitric oxide (NO) being involved in the activation of antioxidant responses and cell survival. However, recent evidences proved that NO-derivatives in proper mixtures with ROS in PTWS could enhance rather than reduce the selectivity of PTWS-induced cancer cell death through the inhibition of specific antioxidant cancer defenses. In this paper we discuss the formation of RNS in different liquids with a Dielectric Barrier Discharge (DBD), to show that NO is absent in PTWS of complex composition like plasma treated (PT)-cell culture media used for in vitro experiments, as well as its supposed protective role. Nitrite anions (NO_2_^-^) instead, present in our PTWS, were found to improve the selective death of Saos2 cancer cells compared to EA.hy926 cells by decreasing the cytotoxic threshold of H_2_O_2_ to non-toxic values for the endothelial cell line.

## 1. Introduction

Non-Thermal Plasmas (NTP) are partially ionized gases consisting of chemically reactive species such as ions, neutrals, free radicals, electrons, UV-VIS radiation, and electric field. Besides natural events, such as the Aurora Borealis, NTP can be easily generated by applying an electric field to a neutral gas. When nitrogen and oxygen feeds are used, NTP generate ROS and RNS. In this case, NTP are often tested for biomedical purposes through the exposure of biological targets directly to NTP [1] or to liquids or solutions enriched both with ROS and RNS (RONS) by means of NTP [2].

Since it has been established that the efficacy of conventional anti-cancer therapies, chemo- and radiotherapy, actually relies on a release of ROS in cancer cells, PTWS have been widely tested as novel anti-cancer ROS-generating systems, and proved to cause the selective damaging of cancer cells in vitro [3,4,5,6], but also a significant inhibition of tumors growth in vivo [7,8]. The mechanism of PTWS-induced cancer inhibition, however, still needs to be fully elucidated [9,10,11]. So far it is known that the most stable and abundant ROS and RNS in PTWS are H_2_O_2_, NO_2_^−^, and nitrate (NO_3_^−^) ions [12,13]; plasma phase reactions, as well as cross reactions among RONS and liquid components, however, can also produce short-lived molecules such as ozone (O_3_), superoxide anion (O_2_^●−^), nitric oxide (^●^NO), and peroxynitrite anion (ONOO^−^) [14,15].

The presence of ROS with a strong oxidative power like H_2_O_2_ has been soon argued as one of the main effectors of PTWS-induced cancer cell death [16]; the cytotoxicity of PTWS, though, proved to be generally higher than simple water solutions of H_2_O_2_ [17].

The biological role postulated for RNS, on the contrary, is controversial. A protective role has been first proposed due to the biological role of nitric oxide. ^●^NO is a unique diffusible molecular messenger in the vascular and immune system involved in neurotransmission [18], stimulation of the immune system [19], and protection mechanisms against pathogens and cancer cells [20]. Many diseases, including cancer, have been associated to restricted ^●^NO bioavailability [21] and ^●^NO donor drugs are nowadays used for many therapeutic purposes [22,23,24]. In the case of strong oxidative stress, it is quite established that ^●^NO can play a protective role [25,26] through the upregulation of protective proteins such as heat shock proteins, cyclooxygenase-2, or heme oxygenase-1 as endogenous cytoprotection [27,28], eventually promoting cancer cell survival. The presence of ^●^NO could thus decrease the potential clinical efficacy of PTWS, due to the antioxidant response induced over cancer cells.

In contrast with this evidence, however, it was also demonstrated that high levels of ^●^NO may induce direct DNA damage, or lead to the formation of the highly oxidative peroxynitrite ONOO^−^ [22,29], which, in turn, can damage lipids, DNA, and proteins, addressing cells to necrosis or apoptosis [30]. One of the most important examples of this conversion is the reaction of NO with ROS, especially with O_2_^●−^ [31]. Indeed, there is also evidence that nitric oxide synthases (NOX) on tumor cells membranes can produce O_2_^-^ in addition to NO under stress conditions [30], thus generating ideal conditions for the local formation of ONOO^−^. Under these conditions, therefore, NO was found to strengthen rather than decrease the oxidative stress induced by plasma-generated ROS on cancer cells [29,30]. Regarding this topic, Kamm et al. recently discussed the importance of even exogenous NO-derivatives, nitrites and nitrates, as an alternative way to produce intracellular bioactive ^●^NO, to inhibit cancer proliferation and induce cancer cell death [31]. Furthermore, recent evidence proved the enhancement of selective cancer cell death due to a synergic action of NO-derivatives with oxygenated species also through PTWS [32,33].

Therefore, a double-edge role of RNS as protectors and inducers of oxidative stress emerged. The control of intracellular RNS may thus offer a sophisticated strategy to tune the anticancer effects of RONS-based therapies, including PTWS. To carefully balance the composition of PTWS, however, a deep knowledge of the chemical routes of formation for each species is required.

To date, this knowledge has not been fully achieved. The lack of reliable detection methods of RONS in complex matrices like PTWS, is currently the biggest limitation. Due to the complexity of the study, most of biological effects were reported for PTWS produced by plasma treatment of cell culture media (i.e., liquid media containing carbohydrates, vitamins, growth factors, amino acids, and inorganic salts) to be used in vitro [11,34,35,36,37] while, for sake of simplicity, most of the chemical studies investigating RNS formation in PTWS have been performed in simple model liquids like water or similar [38]. With specific regard to ^●^NO, however, chemical routes of formation in PTWS seems to be strongly affected not only by the environment where the plasma is ignited, but also by the chemical composition of the liquid to be treated. In general, the presence of the organic compounds required to improve the biological compatibility of PTWS not only increases the complexity of the study but provides alternative routes for the suppression or promotion of plasma-diffused ROS or RNS in the liquid. Chemical studies, extensively analyzing the strategies for modulation of RNS species even in PTWS of complex composition, are therefore of extreme importance.

The aim of this paper is to investigate the capability of NTP to generate ^●^NO and its derivatives such as NO_2_^−^ ions in different liquids exposed to a DBD plasma source, with the purpose to better understand the supposed biological role of RNS in PTWS and in promoting anti-cancer effects. For this purpose, Dulbecco Modified Eagle Medium (DMEM), a cell culture medium containing more than 30 organic components (vitamins, carbohydrates, amino acids, antibiotics, and inorganic salts) is here used for the first time to investigate routes of formation of the most important RNS in comparison with water.

Besides the composition of the liquid target, the DBD gas feed was tuned from O_2_ to synthetic air, and N_2_ in order to demonstrate how plasma composition can be programmed so as to, respectively, exclude or promote the presence of RNS species (i.e., NO_2_^−^) in PTWS synthesized from DMEM. RNS-enriched and RNS-free PTWS were then used for the incubation of osteosarcoma cancer cell lines (i.e., Saos2) and hybrid endothelial cells (i.e., Ea.hy926 cells), and for ultimately confirming that NO-derivatives increase the extent and selectivity of ROS-induced death of Saos2 cells with respect to the endothelial EA.hy926 ones.

Most tumors are characterized by an inflammatory microenvironment, and correlations between inflammation and cancer progression have been extensively reported in literature [39,40,41]. It is well established that cancer cells can fuse with endothelial cells to form hybrid cells spontaneously, which facilitates cancer cells traversing the endothelial barrier to form metastases [42]. However, up to now, little is known about the biologic characteristics of hybrid cells. Endothelial cells (EC), as part of the tumor microenvironment, play a crucial role in inflammatory processes, as well as in angiogenesis and could be critical targets of cancer therapy like, as an example irradiation [43], and as in our case, exposure to plasma treated water solutions. For this reason, our work is aimed to assess whether a different behavior is observed for two cell lines used together to represent a study model of cancer, the Saos2, and hybrid endothelial cells, Eahy926, with tumor mass-vessel wall characteristics. Eahy926 cell line was derived from fusion of human umbilical vein endothelial cells with human lung adenocarcinoma cell line A549. A study published in 2009 [44] showed that in this cell line the proliferation ability was similar to that of A549 cells, but the ability in adhesion and migration of Eahy926 cells was higher. In addition, Eahy926 cells had weaker ability of invasion and could not form tumor mass. These characteristics led us to use them as an experimental model to compare with the Saos2 cancer cell line. A recent paper shows that ECs, as part of the inflammatory microenvironment of tumors, are important regulators of the actual tumor response to radiation therapy [43]. Moreover, EAhy926 have been used as model cells to assess whether cordycepin regulates proliferation, migration and angiogenesis in the hepatoma [45]. Following these features we used Saos2 and EAhy926 cell lines as cell model to gain new insights in the role of RNS in combination with ROS against cancer. Our results, therefore, indicate how PTWS could be a powerful and sophisticated tool to balance RNS- and ROS-induced cellular stress in cancer treatment applications, and support the emerging role of NO-derivatives mediated by PTWS as inducers rather than protectors of ROS-induced oxidative stress in cancer cells.

## 2. Materials and Methods

### 2.1. Plasma Setup and PTWS Generation

**Figure 1:**
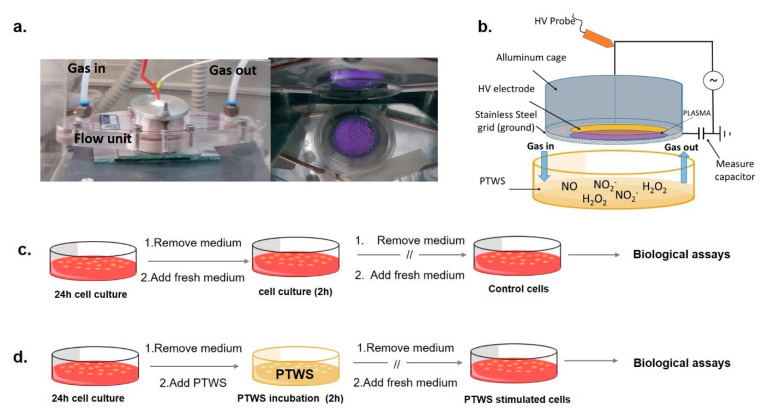
Experimental apparatus and scheme of the biological testing (**a**): left, Plexiglas chamber containing the modified Petri Plas+ plasma source; right, bottom-view of the plasma in air gas feed. (**b**) scheme of the source facing a Petri dish containing the liquid to be treated. After 24 h of cell growth, cells were incubated with 2 mL of (**c**) untreated medium (in case of control samples) and (**d**) Plasma Treated-DMEM (PT-DMEM, in case of PTWS stimulated cells) for 2 h at 37 °C, 5% CO_2_. After, the liquid was replaced with a cell culture medium, and cell cultures were prolonged until biological assays.

Cold atmospheric plasmas were generated in a DBD PetriPlas^+^ source, engineered by the Leibniz Institute for Plasma Science and Technologies (INP, Greifswald, GER), and modified by co-authors of this paper. The source consists of a Plexiglas flow unit set with gas connections, sockets, and a discharge unit (Figure 1a), as schematized in Figure 1b.

A full description of the experimental apparatus has been provided in our previous papers [5]. An electric field of 6 kHz frequency and peak-to-peak voltage of 13 kV was applied with a power supply connected to a programmable 10 MHz DDS (Direct digital synthesis) function generator (TG1010A, Aim-TTi, Huntingdon, UK) to ignite a volume discharge as wide as the HV electrode (Figure 1a).

Plasma treatments were performed on a commercial TPP^®^ Petri dish (57 mm diameter, Techno Plastic Products (TPP), Trasadingen, CH) containing 2 mL of liquids, positioned beneath the source, at a distance between the liquid and the ground grid of 3 mm. A closed system is set in this way, whose chemical composition can be properly conditioned by purging it with the gas feed before igniting the plasma. High glucose DMEM (catalognumberD1145, Sigma–Aldrich, St. Louis, MO, USA) without phenol red, supplemented with 10% *v*/*v* Fetal Bovine Serum (FBS, cat. N. F7524 Sigma–Aldrich), 2mM L-glutamine (cat. N. G7513, Sigma–Aldrich), penicillin and streptomycin solution (20 units mL^−1^/20 mg mL^−1^, T4049, Sigma–Aldrich) was used for plasma treatments and, when untreated, as a reference in all biological experiments; for Electron Paramagnetic Resonance (EPR) analyses also serum free DMEM was used to assess the ability of components of DMEM to scavenge ^●^NO; water and water solutions were also used for the EPR experiments. The energy dose delivered during the discharges was calculated with the voltage–charge (i.e., the time integral of the current) Lissajous method described in our previous works [5,6]. Data are summarized in Table 1.

### 2.2. Detection of ^●^NO in PTWS

**Figure 2:**
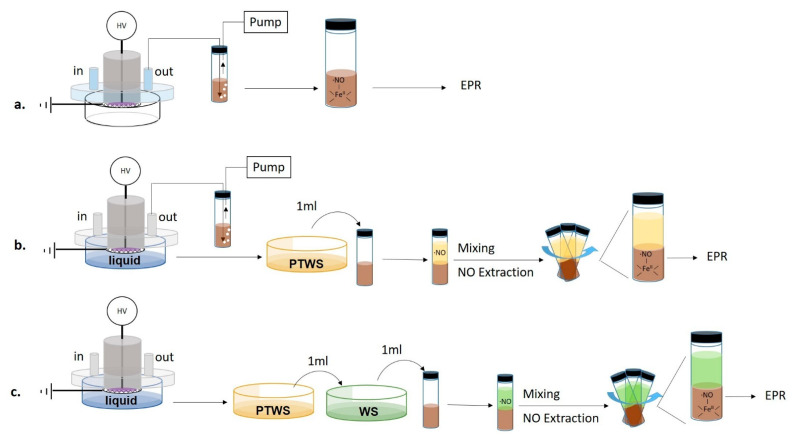
Scheme of the experimental procedures used for the Electron Paramagnetic Resonance (EPR) detection of nitric oxide. (**a**) Detection of nitric oxide (^●^NO) in plasma effluents without the liquid (**b**) Contemporary detection of ^●^NO in plasma effluents and PTWS. (**c**) Detection of ^●^NO in PTWS after mixture with untreated Water Solutions (WS) before the trapping step.

EPR measurements were carried out using an iron(II) N, N-diethyldithiocarbamate, Fe^2+^(DETC)_2_ spin trap, synthesized by mixing 25 mL of a water solution containing 2 × 10^−2^ M of diethyldithiocarbamate (DETC) with 100 mL of FeSO_4_∙7H_2_O (10^−2^ M in water) for 60-min. The mixture was stirred for 2 h, and the precipitate Fe^2+^(DETC)_2_ was collected by filtration. All processes were run in an N_2_ atmosphere. Since the spin trap is soluble only in organic solvents, in all EPR experiments a spin trap solution in dichloromethane (CH_2_Cl_2_, cat. N. 270997, Sigma–Aldrich), from now on named “trap solution”, was used. PTWS or plasma effluents were put in contact with the trap solution by feeding the DBD chamber with N_2_. The production of ^●^NO in plasma phase was assessed by bubbling the gas effluents from the DBD directly in the trap solution for the entire duration of the plasma treatment. The sampling of the plasma effluents was carried out both with and without the liquid into the Petri dish (Figure 2a,b).

To analyze the ^●^NO production in the liquid, 1 mL of PTWS was added to 1 mL of trap solution and mixed to promote the extraction of ^●^NO from PTWS (Figure 2c).

^●^NO was also measured in PTW, NO_2_^−^- water solution (170 µM, prepared by sodium nitrite, Sigma–Aldrich ≥ 99.0%); pH = 3), plasma-treated cysteine (PT-Cys) solution (0.006 g L^−1^, prepared from cat. N. C8755, Sigma–Aldrich), plasma-treated glucose (PT-Glu) solution (45 g L^−1^ solution, prepared from D-(+)-Glucose solution 45% in H_2_O, Sigma–Aldrich), and Plasma Treated DMEM (PT-DMEM). The same solutions, untreated, were used as reference (DMEM). In some cases, such liquids were mixed with other PT- or untreated water solutions (WS) as described in the results section. In all mixing experiments, 1 mL of starting liquid was mixed with 1 mL of the WS to be tested, then 1 mL of the mixture (starting liquid+ WS) was mixed with 1 mL of the spin trap.

The samples to be analyzed were transferred to a glass capillary and inserted into the cavity of a 9-GHz Varian E-9 Century Line EPR spectrometer. Instrumental settings were: 12 mT scan width (3000 points, for a spectral resolution of 0.004 mT per point); 100 kHz and 0.16 mT field modulation frequency and amplitude, respectively; 9.5 GHz and 15 mW microwave frequency and power, respectively; 0.064 s time constant; 8 min scan time; room temperature.

As a method alternative to EPR, a spectrofluorometric assay with 4-Amino-5-Methylamino-2′,7′-Difluorofluorescein Diacetate (DAF-FM-DA, cat. N. D2384, Thermo Fisher Scientific, Waltham, MA, USA) was used to assess the presence of ^●^NO in PTWSs. DAF-FM was obtained by basic hydrolysis of DAF-FM-DA [46]. Spectrofluorometric analyses were performed with a Fluorolog-3 spectrofluorometer (Horiba Jobin-Yvon, Kyoto, JAP) equipped with a 450 W Xe lamp exciting source, double grating excitation and emission monochromators, and a TBX single-photon counter detector. Optical measurements were performed at room temperature; samples were analyzed immediately after incubation with DAF-FM at the excitation wavelength of 450 nm. Positive controls for ^●^NO in water and DMEM were assessed by using the ^●^NO donor Diethylamine NONOate diethyl ammonium salt (DETA-NONOate, cat. N. D185, Sigma–Aldrich). Solutions of DETA-NONOate (100 µM) in DMEM and water were used 2h after the preparation.

### 2.3. H_2_O_2_ and NO_2_^−^ Detection in PTWS

The colorimetric detection of H_2_O_2_ and NO_2_^−^ ions was performed soon after the plasma treatment. H_2_O_2_ was detected with a copper-phenanthroline assay (Spectroquant^®^, cat. N. 118789, Merck Millipore, Burlington, MA, USA). Ions NO_2_^-^ were detected with the Griess assay (Spectroquant^®^, cat. N. 114776, Merck Millipore, Burlington, MA, USA). UV-Vis absorbance measurements were performed with a Cary 60 UV-Vis (Agilent, Santa Clara, CA, USA) spectrophotometer. Details about data processing are described in the supporting information.

### 2.4. Biological Assays

The biological effects of PT-DMEM with different ROS–RNS ratios were analyzed after cell incubation for 2 h (37 °C, 5% CO_2_), according to the schemes in Figure 1c,d.

The cytotoxic effect of different PT-DMEM was evaluated with the Saos2 cell line (American Type Culture Collection, ATCC, HTB-85), chosen as a model for cancer cells, and EAhy926 (ATCC, CRL-2922), chosen as a model for endothelial cells. Both cell lines were cultured in high glucose DMEM supplemented with 10% of FBS in a humidified atmosphere containing 5% CO_2_ at 37 °C. About 20 h before the plasma treatment, cells were seeded in 57mm diameter TPP^®^ tissue culture Petri dishes with a density of 10^4^ cells per dish. There were 3 samples per each PT-DMEM and per each growth time were used. After that, the growing medium was removed and replaced with 2 mL of PT-DMEM. For control cells, the original medium was replaced with 2 mL of untreated fresh medium. After 2 h of incubation (time 0 h), PT-DMEM was removed, cells were rinsed once with untreated DMEM, and left growing for 24 and 72 h.

After 24 and 72 h cells were stained with a Coomassie Brilliant Blue solution, as described in our previous papers [6,37]: cells were fixed with 4% paraformaldehyde in Phosphate Buffer Saline (PBS) for 20 min, rinsed twice with PBS, then a Coomassie Brilliant Blue (R250, Biorad, Hercules, CA, USA) solution in methanol (45% *v*/*v*), acetic acid (10% *v*/*v*), and water (45% *v/v*) was added for 3 min. After rinsing with double distilled water (ddH_2_O), cells were observed using an inverted optic microscope (Nikon Eclipse Ti); at least 7 pictures per time/type/dish were acquired with a Nikon DS Fi2 CCD camera. Image J analysis was performed to calculate the percentage of the substrate area covered by the cells, described as cell density in the Results Section.

### 2.5. Statistical Analysis

Statistical differences among groups, at least 3 replicates per group, were determined by one-way or two-ways-ANOVA assuming normal distribution of data, followed by Tukey’s or Bonferroni’s post-hoc tests.

## 3. Results

### 3.1. Detection of Plasma Produced Exogenous ^●^NO in Gas and in PTW

**Figure 3:**
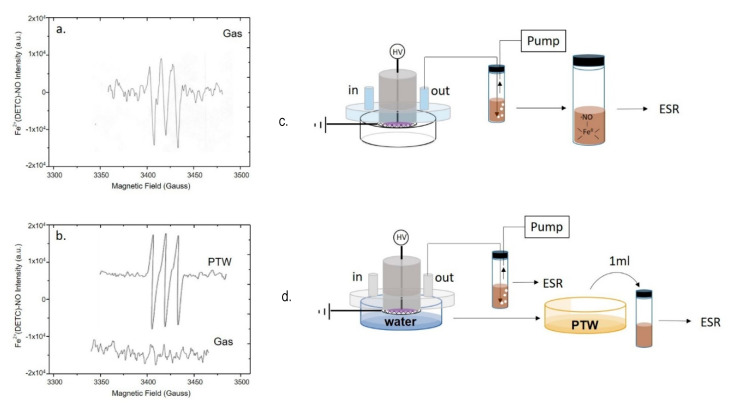
Production of ^●^NO in the plasma phase or in the liquid underneath. EPR spectra of mononitrosyl-iron complex MNIC acquired by (**a**,**c**) bubbling the plasma effluent in the spin trap solution during a DBD with no liquid present, or by (**b**,**d**) simultaneous sampling the plasma effluent and the PTW. Experimental DBD conditions: 15 min, 0.5 (SLM) Air, 13 kV, 6 kHz, 50% DC, 100 ms period. The experimental schemes used for EPR detection are reported on the right of each spectrum.

EPR analyses were carried out to detect plasma generated ^●^NO through the formation of a mononitrosyl-iron-dithio-carbamate (MNIC), NO-Fe^2+^(DETC)_2_ as spin trap for the radical. Spectra were acquired directly from the plasma effluents either during the DBD with no liquid present (Figure 3a), or in the presence of ddH_2_O underneath the discharge (Figure 3b). To reveal ^●^NO in the plasma, the analysis was carried out by bubbling plasma effluents into a vial filled with the spin trap solution and connected to the pump (Figure 3a).

The EPR spectrum shows a single triplet due to the adduct of ^●^NO to the trap, which was identified as the paramagnetic five-coordinate complex NO-Fe^2+^(DETC)_2_, characterized by a hyperfine coupling constant of a_N_ = 13.10 G, and g-factor = 2.039.

When the same discharge was carried out in presence of ddH_2_O in the Petri dish the EPR signal of ^●^NO, is revealed only in the liquid (Figure 3b, PTW), but not in the plasma effluents (Figure 3b, gas). The PTW sample was also tested using a spectrofluorometric assay based on the DAF-FM NO probe; the fluorescence intensity emitted from DAF-FM consists of a signal with a maximum at 515 nm attesting the presence of ^●^NO and its derivatives in PTW, in agreement with the EPR, as reported in Figure S1.

### 3.2. Plasma Generation of Exogenous ^●^NO in PT-DMEM and PTWS Containing Organic Components

**Figure 4:**
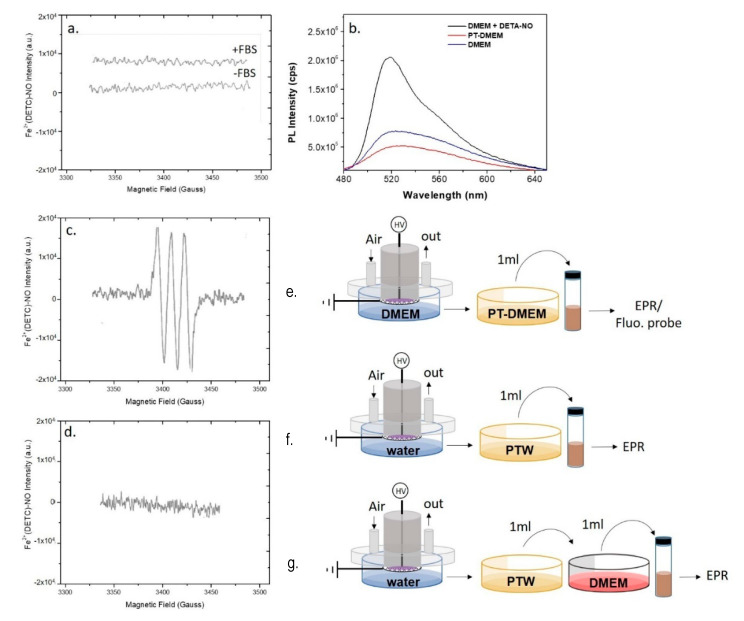
Plasma synthesis of ^●^NO in ddH_2_O and DMEM. EPR spectra of (**a**,**e**) PT-DMEM with (+FBS) and without (-FBS) Fetal Bovin Serum (FBS), (**c**,**f**) PTW, and (**d**,**g**) PTW mixed with 1 mL of untreated DMEM. (**b,e**) Fluorescent spectra of PT-DMEM, DMEM with the addition of the ^●^NO donor DETA-NONOate (100 µM) and untreated DMEM (NT-DMEM) after the use of the fluorescent probe DAF-FM excited at 450 nm All measurements were acquired 15 min after the DBD. Experimental conditions: 15 min, 0.5 slm Air, 13 kV, 6 kHz, 50% DC, 100 ms period. Schemes of the experimental procedures used for the EPR are reported on the right of each spectrum.

PT-DMEM with and without serum was analyzed by EPR similarly to PTW; the resulting spectra are reported in Figure 4. In the same experimental conditions allowing the detection of ^●^NO in PTW, no EPR signal was observed in PT-DMEM (Figure 4a). This was also confirmed with the DAF-FM assay, where almost no changes of the fluorescence intensities were found between PT and untreated DMEM both with and without serum (Figure 4b, Figure S4). To exclude possible interferences of DMEM components, the ability of the fluorescent probe to detect ^●^NO in DMEM was confirmed by using a solution of the ^●^NO donor DETA-NONO in DMEM (100 µM) as a positive control (Figure 4b, Figure S4).

By assuming that ^●^NO is formed in the plasma phase and then diffuses into the liquid, since ^●^NO is not detected in the liquids we assume that some chemical component or conditions (e.g., the buffered pH) in DMEM are responsible for the abatement of the plasma diffused ^●^NO in the treated medium. In Figure 4a is reported the spectrum acquired on DMEM demonstrating the absence of the MNIC signal both in case of DMEM with and without serum. On the other hand, it was found that the addition of untreated DMEM to PTW suppresses the strong EPR signal acquired in PTW (Figure 4c,d), attesting for the presence of components of the serum free medium able to scavenge ^●^NO. Among all DMEM components, L-cysteine and D-glucose were identified as possible scavengers of ^●^NO; L-Cysteine, for its ability to form nitrosothiol groups [47], D-glucose, because it is the most abundant component of DMEM (4.5 g L-1). To ascertain whether these two compounds scavenge or not ^●^NO, their solutions at the same concentrations as in DMEM were plasma treated in the same conditions as for PT-DMEM and PTW, and named PT-L-cysteine and PT-D-glucose, respectively. PTWS were not buffered, to show similar conditions as PTW.

**Figure 5:**
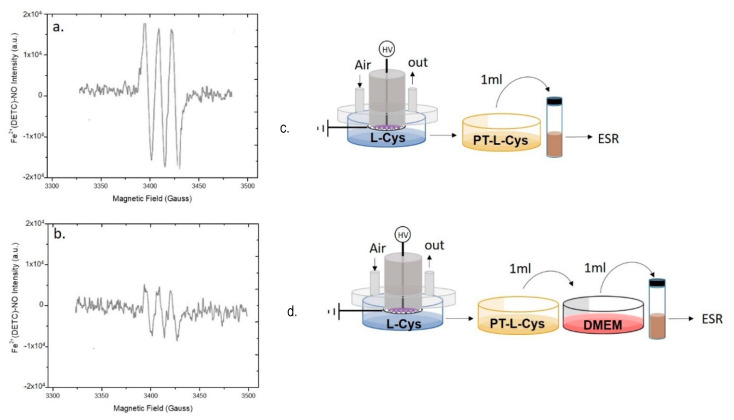
Role of Cysteine in scavenging plasma produced ^●^NO. EPR spectra of (**a**,**c**) a Plasma Treated L- cysteine (PT-L-cysteine) solution (0.006 g L^−1^), and (**b**,**d**) PT-L-cysteine solution mixed with untreated DMEM acquired 15 min after the discharge. Experimental conditions: 0.5 slm Air, 50% DC, 100 ms period, 6 kHz, 13 kV, 2 mL solution, 15 min treatment time. Schemes of the experimental procedures used for EPR analysis are reported on the right of each spectrum.

The EPR spectrum of PT-cysteine (Figure 5) shows the MNIC signal; this limits or excludes the role of cysteine as ^●^NO scavenger in our conditions. Moreover, when PT-L-cysteine was mixed with DMEM (Figure 5b), the signal was not suppressed as in the case of DMEM added with PTW (Figure 4d). In particular, by comparing the intensities of the spectra (Figure 5a,b) it can be observed that when PT-cysteine was mixed with untreated DMEM the intensity of the signal decreased but did not disappear, likely due to some of the DMEM components capable of scavenging ^●^NO.

**Figure 6:**
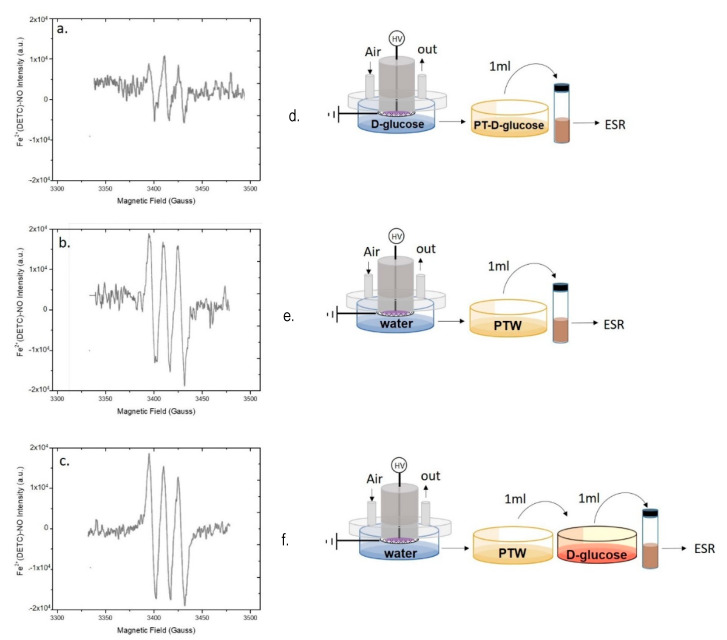
Role of D-Glucose in scavenging plasma produced ^●^NO. EPR spectra of (**a**,**d**) Plasma Tretaed- D-glucose (PT-D-glucose) solution (45 mg L^−1^), (**b**,**e**) PTW, and (**c**,**f**) PT-D-glucose solution mixed with PTW 15 min before spectra acquisition. Experimental conditions: 0.5 slm Air, 50% DC, 100 ms period, 6 kHz, 13kV, 2 mL solution, 15 min treatment time. Schemes of the experimental procedures used for EPR analysis are reported on the right of each spectrum.

The EPR spectra acquired on PT-D-glucose are reported in Figure 6. Although the MNIC EPR signal is present in PT-D-glucose (Figure 6a), its intensity was lower than in PTW (Figure 3b and Figure 4c), while in the case of PT-cysteine, the signal was similar to that in PTW (Figure 5a).

This decrease suggests that D-glucose contributes a bit to scavenge ^●^NO in PT-DMEM, but the persistence of the signal does not allow to identify D-glucose as the only cause of ^●^NO abatement in this liquid. Moreover, when PTW is mixed with the untreated solution of D-glucose, the intensity of the EPR signal was not decreased (Figure 6c). This last result seems to attest that PT-D-Glucose has some ^●^NO scavenging ability, likely due to some derivative of D-Glucose after the plasma treatment, while untreated D-Glucose has not.

### 3.3. Plasma Generation of Exogenous H_2_O_2_ and NO_2_^−^ in PT-DMEM

**Figure 7:**
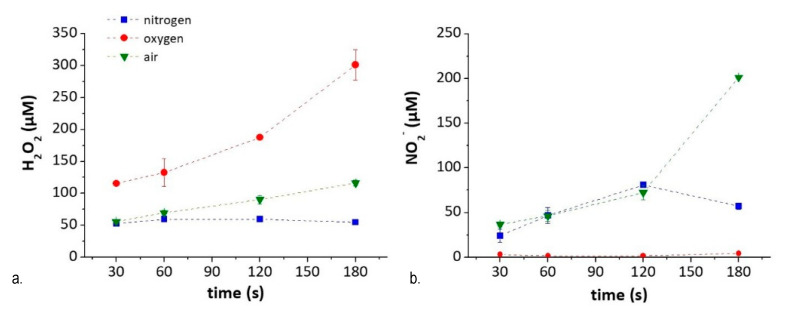
Chemical composition of PTWS as a function of experimental conditions- Content of (**a**) H_2_O_2_ and (**b**) NO_2_^−^ in PT-DMEM according to feed composition and treatment time.

From the experiments described so far, it is clear that the plasma-induced generation of ^●^NO in DMEM is scavenged by derivatives of D-Glucose generated after the treatment, and by some other compound. The possibility to produce other RNS, such as NO_2_^−^, in PT-DMEM was therefore tested, by exploring plasma parameters affecting their production. O_2_, N_2_, and synthetic air were used as gas feed to exclude or promote the presence of RNS in PT-DMEM, respectively, and to relate it to the amount of ROS generated. We decided to analyze the concentration of NO_2_^−^ and H_2_O_2_ used as markers, respectively, of all RNS and ROS species present in the PT-DMEM due to their estimated long life-time also in complex media. The presence of different species like as an example ONOO^−^ and O_2_^●−^ cannot be in any case excluded [37]. The results of the chemical composition of PT-DMEM in terms of NO_2_^−^ and H_2_O_2_ concentration after N_2_, O_2_^●^− and air-DBDs, performed at different treatment times (30–180 s), are reported in Figure 7. As shown in the graphs, the treatment time affects the amount of the species produced rather than the kind, that is determined only by the composition of the gas feed.

H_2_O_2_ (i.e., marker of ROS species) in PT-DMEM increases with the percentage of O_2_ in the feed, from N_2_ (where the source of oxygen in the feed comes from water evaporated from the liquid) to pure O_2_. The NO_2_^−^ (i.e., marker of RNS species) concentration in the liquid, instead, is higher when both O_2_ and N_2_ are fed in the plasma, as in the case of air-treated DMEM, and decreases in liquids treated with N_2_-DBD. For the same reason, no NO_2_^-^ is found in PT-DMEM obtained by O_2_-DBDs, due to a purging procedure that eliminates any remaining air at the plasma–liquid interface (see Methods).

The possibility to achieve such fine control over the PT-DMEM composition revealed to be decisive to ascertain the role of NO_2_^−^ and other undetected RNS species as stress effector, synergic with ROS, to cells exposed to PTWS. PT-DMEMs with different H_2_O_2_–NO_2_^−^ ratios, as listed in Table 2, have been thus easily produced for cytotoxicity experiments. We decided to name the different PT-DMEM according to their H_2_O_2_ and NO_2_^-^ contents, as listed in Table 2.

### 3.4. Cells Incubation with PT-DMEM Containing Different Exogenous H_2_O_2_-and-NO_2_^−^ Doses

**Figure 8:**
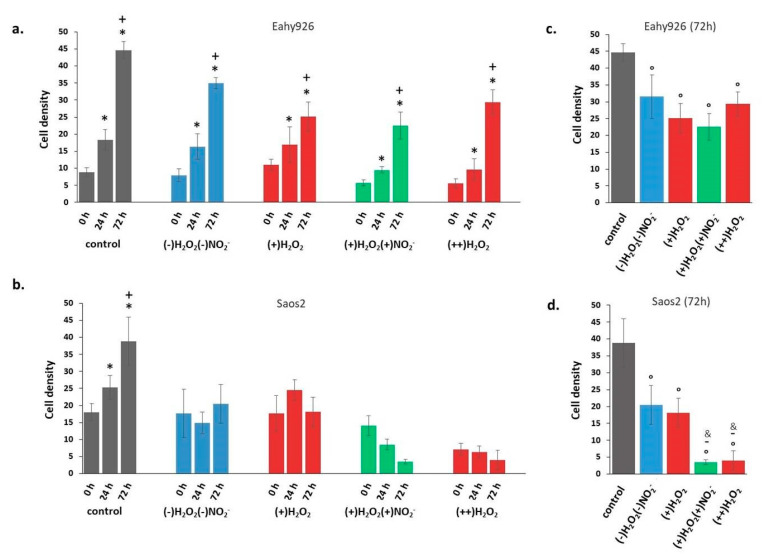
Cell density of cells incubated with PTWS respect to the control. (**a**) Eahy926 and (**b**) Saos2 cells at different time points (0, 24, and 72 h) after incubation in different PTWSs. (**c**) Eahy926 and (**d**) Saos2 cells after 72 h of incubation in different PTWSs. The time 0 h corresponds to cells exposed for 2h to PTWSs, PFA4% fixed, rinsed with PBS and Coomassie blue stained. One-way ANOVA + Tukey’s post-test: (*) = *p* < 0.01 vs. 0 h; (+) = *p* < 0.01 vs. 24 h; (°) = *p* < 0.01 vs. control; (-)= *p* < 0.01 vs. (+)H_2_O_2_; (&) = *p* < 0.01 vs. (-)H_2_O_2_ (-)NO_2_^−^.

The modifications of the density of Saos2 and EAhy926 cells after incubation with PT-DMEMs with different H_2_O_2_–NO_2_^−^ ratios are summarized in Figure 8. Cell density is intended as the area of Petri dish covered by cells, calculated by ImageJ analysis. Some representative images acquired on Coomassie Blue stained cells 72 h after incubation with PT-DMEM at different H_2_O_2_–NO_2_^−^ ratio are shown in Figure S2 and Figure S3.

Cell growth data at different time points, in Figure 8a,b, show that incubation in all PT-DMEM synthesized resulted in no growth for Saos2 cells (Figure 8b). In the case of EAhy926 cells, instead, growth was measured in all considered conditions (Figure 8a). A first selective effect of PT-DMEM as inhibitors of the growth of cancer cell could thus be observed, regardless of the H_2_O_2_–NO_2_^−^ ratio. However, looking at cell densities after 72 h incubation (Figure 8c,d), a specific response of the Saos2 cells can be clearly observed, depending on the H_2_O_2_–NO_2_^−^ ratio in the liquid, differently from EAhy926 at the same time point.

Both cell lines show a reduced density after incubation in PT-DMEM in comparison with control cells; however, in the case of EAhy926 cells (Figure 8c), the different H_2_O_2_–NO_2_^−^ ratio in the liquids did not produce statistically significant differences in cell density. In the case of Saos2 cells, instead, besides a general stronger density decrease in comparison with the control, the specific effect of each liquid changed depending on their specific H_2_O_2_–NO_2_^−^ ratio. A mild effect was produced after incubation in (-)H_2_O_2_, (-)NO_2_^−^, and (+)H_2_O_2_, while the strongest decrease was caused by (+)H_2_O_2_, (+)NO_2_^−^, and (++)H_2_O_2_ media. The anti-cancer effects of NO_2_^-^ emerged, in particular,: the simultaneous presence of H_2_O_2_ (about 120 µM) and high doses of nitrite ions (about 200 µM), as in (+)H_2_O_2_, (+)NO_2_^-^, decreased Saos2 cell density much more than the same amount of H_2_O_2_ alone, as in (+)H_2_O_2_, whose effect, conversely, was quite mild. When Saos2 cells were exposed to (++)H_2_O_2_ solutions for 2 h (0 h in Figure 8b), before 4%PFA/PBS fixation, round cells (probably dead cells) floating in the liquid were observed attesting for the deleterious effect of such solutions since first hours of observation.

**Figure 9:**
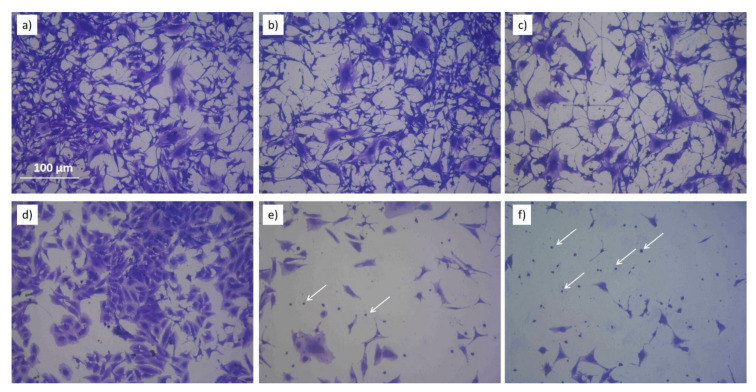
Coomassie Blue images of Eahy926 (**a**–**c**) and Saos2 (**d**–**f**) cells incubated with PTWS and let grown for 72h. Cells no incubated with PTWS are shown in (**a**,**d**). (**b**,**e**) show cells incubated with (−)H_2_O_2_ (−)NO_2_^−^. (**c**,**f**) show cells incubated with (+)H_2_O_2_ (+)NO_2_^-^. White arrows in images (**e**,**f**) show spherical Saos2 cells.

Morphological changes in EAhy926 and Saos2 exposed to PTWS with different ROS/RNS and stained 72h after the exposure can be, respectively, appreciated by looking at Figure 9 that shows pictures of Comassie blu stained cells (higher magnification than the ones shown in Figure S2 and Figure S3). In case of the endothelial cell line, the morphology of the cells is almost retained after the treatment with PTWS. Looking at single cells (Figure 9), it is possible to observe the retention of the typical starry shape of this cell line; while, by analyzing the overall appearance of cells, the PTWS exposed ones do not remarkably differ from control cells for cell density on the substrate and for the capability to form clusters among each other, another important feature of endothelial cell lines (Figure S2). Endothelial cells show only a change on cell density and not in morphology, differently from what has been observed for Saos2 cells. In case of Saos2 cells, in fact, the morphology of PTWS-exposed cells is dramatically impacted. It is evident that a loss in cell clustering is present in all the Saos2 cells incubated with PTWSs. Furthermore, where the cells show the less density also a predominance of spherical cells are present, clear evidences of apoptotic or pre-apoptotic conditions (white arrows of Figure 9 e,f). Then, it is possible to observe the loss of the typical stretched shape of this cell line, in favor of a more condensed form which is generally associated to a stressing environment. These changes gradually occur depending on the specific chemical composition of the tested PTWS: Saos2 cells incubated with (−)H_2_O_2_(−)NO_2_^−^ show a milder reduction in cell density and only a partial degradation of cell shape, while cells incubated with (+)H_2_O_2_(+)NO_2_^−^ show a radical change in morphology, with small, spherical and isolated cells being greatly reduced in number.

## 4. Discussion

Understanding the dual role of RNS as both protectors and inducers of oxidative stress is of peculiar importance for controlling intracellular RNS and, ultimately, for tuning anticancer effects of RONS-based approaches and therapies, including those based on PTWS [48]. However, the generation of PTWS with predetermined chemical composition for a specific target requires a deep knowledge of the chemical formation routes of each bioactive specie in the plasma-activated liquid. This knowledge, to date, is highly desired but still far to be achieved, above all in the case of those PTWS generated from bioactive liquids with a complex chemical composition, that would typically be required for in vitro experiments (i.e., cell culture media), or for in vivo administration (i.e., blood, biological fluids). Our study is therefore aimed at clarifying, from one side the routes of formation of RNS in PTWS generated in bioactive liquids like DMEM, and, on the other side, at demonstrating how the modulation of RNS concentrations in PT-DMEM used incubation with cells effectively concurs to tune anticancer effects.

The chemical analysis performed to assess the presence of ^●^NO in water and in DMEM with (+FBS) and without serum (-FBS) after plasma treatment confirmed that the formation of ^●^NO strongly depends on the composition of the liquid to be treated. Although ^●^NO was present in plasma effluents and in PTW (Figure 3a), no trace of ^●^NO was found in PT-DMEM (+/− FBS), due to its scavenging in part by D-Glucose and by other compounds still to be identified. It is not excluded that the serum could additionally contribute to scavenge ^●^NO, due to its content of organic molecules. However, as well known, the FBS is a not fully defined medium and as such, may vary in composition between batches, thus it is quite difficult to identify the molecule of FBS responsible of such scavenging. In any case, the absence of dissolved free ^●^NO in PT-DMEM would exclude the involvement of this RNS during incubation with cells, at least in our conditions, although it cannot be excluded that ^●^NO is first bound by some of the species in the medium, then transferred to cells with no passage through the free solvated form. Also Chauvin et al. [49] could not find trace of ^●^NO after EPR analysis of PT-PBS and PT-DMEM (+/− FBS), and tentatively explained the lack of ^●^NO, that would have been detected at low pH, with the neutral pH of the buffered media investigated. A possible explanation for this evidence, in fact, emerges from the analysis of the production routes of ^●^NO in PTWS, schematically summarized in Table 3.

^●^NO is generally produced in the plasma phase through chemical reactions among N_2_, O_2_, atomic oxygen (O) and nitrogen (N) through the well-known Zeldovich mechanism (Equations (1) and (2) in Table 3), or by oxidation of atomic N by hydroxyl radicals (^●^OH) generated from the dissociation of water vapor from the liquid, if is present during the discharge. The diffusion of plasma-generated ^●^NO into the liquid may be thus supposed as the most likely route for the formation of ^●^NO in PTWS. This hypothesis would even be supported in our data by the disappearance of MNIC signal in plasma effluents, and by its appearance in PTW registered when the discharge is ignited in presence of water (Figure 3a,b). However, the dissolution of ^●^NO from the plasma towards the liquid is highly discouraged by the low solubility of ^●^NO in water media (Henry constant, K_H_ = 0.046 at 25 °C [56]) and by the short diffusion distance reported for ^●^NO during plasma treatment of liquids (<1 mm) [54], quite lower than the distance between the liquid and the discharge in our conditions (3 mm).

For these reasons, it is rather believed that the presence of ^●^NO in PTWS may be more likely due to reactions among other plasma-generated RONS that can more easily diffuse in water media, such as NO_2_ (K_H_ = 0.978 at 25 °C) [56], HNO_2_ (K_H_ = 1198 at 25 °C), and ^●^OH radicals (K_H_= 733 at 25 °C) [57]. NO_2_ is generally produced in plasma effluents due to ^●^NO oxidation with oxidants like atomic oxygen (O) or ozone in presence of a third collision body (M) (Equations (4) and (5), Table 3), while HNO_2_ could be formed in the plasma from reactions between ^●^NO and ^●^OH (Equation (6), Table 3). The diffusion of HNO_2_ in the liquid to form NO_2_^-^ that, in turn, can disproportionate to produce ^●^NO (Equation (7) in Table 3), indeed, is the most cited pathway for the formation of ^●^NO in PTWS in the literature [50,53]. However, besides its very low rate constant (k = 13.4), the conversion of NO_2_^−^ to ^●^NO requires acidic conditions (pH < 3.5, [50]) and could thus be prevented in buffered media like PT-DMEM. In the end, in presence of liquid, e.g., DMEM, underneath the plasma, even the other possible route to produce ^●^NO though reaction between ^●^OH and ONOO^−^ (Equation (8), Table 3), could be actually prevented since, again, acidic conditions are generally required to form even ONOO^−^ [58] and, by now, the presence of ONOO^-^ in PT-cell culture media has never been confirmed. The neutral pH of buffered media like DMEM, that discourage ^●^NO formation routes, can be therefore addressed as the main explanation for the abatement of ^●^NO in PT-DMEM found with EPR and spectrofluorometric analysis (Figure 4a,b).

However, the suppression of the MNIC signal in PTW after the addition of untreated DMEM (Figure 4d) also suggests the possible presence of chemical ^●^NO scavengers in the cell culture medium. In the last case, even the low amounts of plasma-generated ^●^NO that manage to diffuse in the liquid could be rapidly consumed in the medium. The starting composition of DMEM includes several components that can react with plasma-produced RONS, some of them may be particularly keen to react with ^●^NO, such as L-cysteine and D-glucose. L-Cysteine is often reported as an “in vivo ^●^NO modulator” through the reaction of its thiol group with ^●^NO [56,59,60,61]. Moreover, as recently reported by Lackmann et al. [51], covalent modifications of cysteine treated with different plasmas have been shown. D-glucose is also actively involved in the scavenging of ^●^NO in many natural processes [62]; it has been found that the exposure of human endothelial cells to the same level of glucose as in the plasma of diabetic patients results in a significant blunting of ^●^NO responses [62].

The EPR spectrum acquired from PT-cysteine (Figure 5a), however, shows the MNIC signal as intense as in PTW, thus excluding cysteine as a possible scavenger for ^●^NO, in our experimental conditions. Although the formation of S-nitrous-cysteine derivatives is likely to occur, the amount of ^●^NO produced in the treatment, or immediately after, is probably much higher than that consumed by cysteine. Very interestingly, in contrast with the supposed role of ^●^NO scavenger for such molecule, a role of “^●^NO protector and modulator” could actually be proposed, since, when PT-L-cysteine was mixed with DMEM (Figure 5b), the signal was not suppressed, differently from the case of PTW (Figure 4d). This evidence can be explained by considering that ROS and RNS in PTWS form nitroso-thiol (S-NO) functionalities on cysteine [63]. Since S-NO bond is very unstable in physiological conditions, a ^●^NO release from S-nitrous-cysteine is often reported [63] and could thus contribute to sustain the MNIC signal when PT-cysteine is mixed with untreated DMEM.

On the opposite, a role of “^●^NO scavenger” emerged for D-glucose, albeit quite low. The intensity of the EPR signal in PT-D-glucose solution (Figure 6a) was much lower than that obtained in PTW (Figure 6b), suggesting that D-glucose contributes to suppress the signal in PT-DMEM. Nevertheless, when PTW was mixed with an untreated D-glucose solution, the intensity of the EPR signal of the resulting solution was not decreased at all (Figure 6c). This evidence seems to be in contrast with the results reported in Figure 6a unless structural modifications of the molecule due to plasma treatment are proposed. Indeed, plasma-induced modifications could probably enhance the reactivity of glucose towards ^●^NO and, as a consequence, its capability to scavenge it. D-glucose, however, cannot be indicated as the unique cause for the MNIC abatement in DMEM, since the signal was still present even at PT-D-glucose with a concentration (45 g L^−1^) 10 times higher than that in DMEM (4.5 g L^−1^), as shown in Figure 6.

We, therefore, can conclude that the absence of ^●^NO in PT-DMEM may be due at least to two concurrent causes, namely, the neutral pH preventing the main formation pathways for ^●^NO, and compounds, such as glucose, able to sequester ^●^NO possibly diffused by the plasma. These evidences undermine the role hypothesized so far for plasma-produced ^●^NO as a protector or inducer of nitrosative stress on cells incubated in PTWS, which must therefore be sought among other RNS such as NO_2_^−^, at least when PTWS are generated in cell culture media. As already mentioned, though, it cannot be excluded that ^●^NO is first bound by some of the species in the medium (e.g., cysteine or others), and then transferred to cells with no passage through the free solvated form.

The presence of NO_2_^−^ anions in PT- culture media, in fact, could act as a source of nitrosative stress as well, due to the biological conversion, internal to cells, in the more reactive species of ^●^NO and ONOO^−^ [6]. Indeed, nitrite ions are considered the largest intravascular and tissue source of ^●^NO in the human body [64]. In turn, nitrite-derived ^●^NO inside cells can be converted to ONOO^−^ as shown above; and, in presence of peroxides such as H_2_O_2_, nitrites can be even directly converted to ONOO¯, thus providing a second route for the formation of this molecule [65] in biological environments.

Recently Bauer [66,67] supposed that anti-cancer effects of PTWS can be due by local reactions of ^●^NO and NO_2_^−^ with H_2_O_2_ near cell membranes These species are implicated in the formation of ONOO^−^ and singlet oxygen ^1^O_2_ near the membrane of cancer cells, leading to local inactivation of the redox enzyme catalase. Tumor cells contribute to their death through further membrane-associated catalase inactivation and reactivation of intercellular apoptosis-inducing ROS and RNS signaling [65].

NO_2_^−^ remain stable many weeks in PTWS generated from culture media, despite the presence of many organic compounds [59,60]. These features thus promote NO_2_^−^ as the ideal reservoir for nitrosative stress in PTWS of complex composition. Moreover, the production of NO_2_^−^ during DBD treatments can be finely tuned by changing conditions, such as treatment time and gas feed. As shown in this paper, the first is important for the amount, while the second for the kind of plasma-generated species in PT-DMEM. Ranging from O_2_-DBD to N_2_- to air-DBD, the presence of NO_2_^-^ in PT-DMEM can be, respectively, excluded or promoted (Figure 7b). In our conditions, H_2_O_2_ formation in PT-DMEM can be promoted by increasing the percentage of O_2_ in the feed, in agreement with the formation mechanism of H_2_O_2_ from OH recombination at the plasma–liquid interface, as discussed in our previous studies [5]. In turn, the NO_2_^−^ concentration in the liquid can be enhanced if both O_2_ and N_2_ are fed in the plasma. The trends obtained are similar to those obtained with a 2D fluid-dynamic model proposed by Verlackt et al. [61], that describes the interaction between a plasma jet and water buffered at pH around 7, as a function of treatment time. The possibility to achieve fine control over ROS and RNS composition in PTWS by our system was revealed to be fundamental for assessing the role of each specific species as effectors in the PTWS-induced stress on cancer cells.

Within the tumor microenvironment, low to moderate levels of ^●^NO derived from cancer and endothelial cells can activate angiogenesis and epithelial-to-mesenchymal transition, promoting an aggressive phenotype [68]. For this reason, to assess what happens when cancer and hybrid ECs are exposed to NOx species, excluding ^●^NO, in combination with ROS (i.e., H_2_O_2_) is fundamental for further application of such liquids in cancer therapy and in general to gain new insights in tumor progression. The higher sensitivity of Saos2 cancer cells to PT-DMEMs with controlled H_2_O_2_–NO_2_^-^ ratio, in comparison with the endothelial EAhy926 cells confirmed that RNS, mainly nitrites, can induce a cell-dependent anti-cancer effect (Figure 8). The key at the base of such selectivity seems to be the decrease in the cytotoxic threshold for H_2_O_2_ in cancer cells, when PTWS with high doses of NO_2_^−^ are used.

From previous experiments it is known that 2 h of incubation in a 300 µM untreated solution of H_2_O_2_ in DMEM (i.e., unexposed to any plasma) is enough to kill Saos2 cells after a few minutes, with this concentration representing a sort of “tolerance threshold” for this cell line (Figure S5). As a consequence, the response of Saos2 cells to PT-DMEM (++)H_2_O_2_ containing about 300 µM of H_2_O_2_ was a strong decrease in cell density, as expected, despite the absence of nitrites. Moreover, it is well known that high doses of H_2_O_2_ can trigger many apoptotic pathways in cancer cells, most of them involving the passage of exogenous H_2_O_2_ through aquaporin (AQP) channels and the direct intracellular damage via Fenton reaction, as discussed by us [6], and by Yusupov et al. [69]. It has also been suggested by Yan et al. [70] that the increased concentration of AQP channels on tumor cells could explain the anti-tumor effects of PTWS.

Besides a higher number of AQP channels, however, cancer cells also specifically exhibit higher amounts of antioxidant defenses that could seize most of the H_2_O_2_ exogenously delivered through PTWS, resulting in the increase in the cytotoxic threshold for most cancer cell lines. This could be the reason why, indeed, the direct H_2_O_2_-apoptosis induction via PTWS is often reported as non-specific for cancer cells [66,71,72], since the high doses of H_2_O_2_ required to overcome membrane-associated catalase on tumor cells could largely exceed the tolerance of endothelial cells too, which lack this protection.

In all these cases, to selectively interact with a specific-redox chemistry on the surface of tumor cells, a mechanism in which NO_2_^−^ cooperates with lower doses of H_2_O_2_ (i.e., lower than the tolerance threshold for endothelial cells) could be more effective to provide specific apoptotic pathways for cancer cells. Indeed, our results are in agreement with those reported by Bauer [65,71,73], where a synergic effect of NO_2_^−^ and H_2_O_2_ was proposed on human KKN-45 gastric carcinoma cells when the H_2_O_2_ concentration was below 160 µM, a threshold for H_2_O_2_-dependent apoptosis for that cancer line. Similar results on the synergic anti-cancer effect of NO_2_^−^ and H_2_O_2_ in PTWS were also reported by Girard et al. on HCT116 human colon cancer cells and Lu1205 human melanoma cells [33], and by Kurake et al. on glioblastoma tumor cells [32], as discussed by Von Woetdtke and Jablonowski [9]. In agreement with these findings by other authors, in this papers it is shown that the selectivity of PTWS-induced cancer cell death relies on a window of H_2_O_2_ concentration where the synergic action of H_2_O_2_ and NO_2_^−^ can induce death in tumor cells but does not affect hybrid endothelial cells. Endothelial cells (EC), as part of the tumor microenvironment, play a crucial role in inflammatory processes, as well as in angiogenesis, and could be critical targets of cancer therapy. In order to promote a deleterious effect contemporary on cancer cells and hybrid endothelial cells as part of the tumor microenvironment, it is necessary to overcome the threshold toxic level for H_2_O_2._ In this way the apoptosis is promoted also in hybrid ECs as attested by the presence of round shaped cells in Figure S3d. In our experience, we have observed that the changes in morphology (reduced numbers of clusters, spherical shape of cells) are important markers of cell reaction to PTWS, while the changes in cell density are representative of the reduction, retention, or increase in cells number (and, indirectly, of their proliferation). Thus we can conclude that ECs have a capability to overcome the oxidative stress differently from that of osteosarcoma cells. This cell susceptibility threshold and the concurrent action of H_2_O_2_ and NO_2_^−^ was supposed by Bauer et al. [65], and the results shown in our paper attest that an exogenous delivery of RNS in proper combination with ROS actually enhances the selectivity of oxidative stress induced in cancer cells. However, much more research has to be done before a clinical application of PTWS will be possible.

## 5. Conclusions

The routes of formation of RNS such as ^●^NO and NO_2_^−^ after DBD plasma treatment of DMEM have been investigated and their involvement in anti-cancer effects on malignant cells was evaluated. A double detection method for ^●^NO was used for the first time to demonstrate that no trace of ^●^NO could be found in PT-DMEM, where, instead, high amounts of NO_2_^−^ are formed. NO_2_^−^ concentration in PT-DMEM revealed to be finely tunable with the plasma operating conditions such as treatment time, feed composition, and composition of the starting liquid. The addition of PT-DMEM loaded with different H_2_O_2_–NO_2_^−^ ratio to cultures of Saos2 and EAhy926 cells allowed to define the contribution of NO_2_^−^ as supporter rather than protector of oxidative stress induced on cancer cells by the H_2_O_2_ ROS present in PTWS. In particular, high doses of NO_2_^-^ were found to increase the selectivity of anti-cancer effects by decreasing the cytotoxic threshold of H_2_O_2_ for cancer cells to unharmful values for hybrid endothelial cell line.

## Figures and Tables

**Table 1:** Energy doses (J cm^−2^) of plasma in DBD ignited in different feed gases (O_2_, N_2_, and synthetic Air with 0.5 slm of flow rate) and different treatment times. The experimental conditions are 13 kV, 6 kHz, and 25% DC with a period of 100 ms on 2 mL of DMEM.

Treatment Time (s)	Energy Dose (J cm^−2^)		
	O_2_	N_2_	Air
30	7.2 ± 0.3	6.12 ± 0.13	6.5 ± 0.7
60	14.5 ± 0.6	12.2 ± 0.3	12.9 ± 1.4
120	28.9 ± 1.3	24.5 ± 0.6	26 ± 3
180	43.4 ± 1.9	36.7 ± 0.8	39 ± 4

**Table 2:** Composition of PT-DMEM (DBDs ignited at 13 kV, 6 kHz, 25% DC, 100 ms period), produced with different feeds (0.5 slm) and treatment times (1 or 3 min) for cell culture experiments.

PTWS	Plasma Condition	H_2_O_2_ (µM)	NO_2_^−^ (µM)
(−)H_2_O_2_(−)NO_2_−	Air 1 min	69 ± 4	46 ± 6
(+)H_2_O_2_	O_2_ 1 min	130 ± 20	1.60 ± 0.08
(++)H_2_O_2_	O_2_ 3 min	300 ± 20	4.3 ± 0.4
(+)H_2_O_2_(+)NO_2_-	Air 3 min	116 ± 4	201.4 ± 1.7

**Table 3:** Reactions representing the possible chemical formation routes of ^●^NO in PTWS.

Reaction	Number	References
N_2_*_(g)_ + ^●^O_(g)_ → ^●^NO_(g)_ + ^●^N_(g)_	(1)	[50,51]
^●^N_(g)_ + O_2(g)_ → ^●^NO_(g)_ + ^●^O_(g)_	(2)	[50,51]
^●^N_(g)_ + ^●^OH_(g)_ → ^●^NO_(g)_ + ^●^H_(g)_	(3)	[50,52]
^●^NO_(g)_ + O_3(g)_ → NO_2(g)_ +O_2(g)_	(4)	[52]
^●^NO_(g)_ + ∙O_(g)_^●^ +M_(g)_^●^ → NO_2(g)_ +M_(g)_	(5)	[52]
^●^NO_(g)_ + ^●^OH_(g)_ → HNO_2(g)_ → HNO_2(aq)_	(6)	[52]
3HNO_2(aq)_ → 2^●^NO_(aq)_ + HNO_3(aq)_ + H_2_O	(7)	[52,53]
^●^OH_(aq)_ + ONOO^-^_(aq)_ → O_2(aq)_ + OH^-^_(aq)_ + ^●^NO_(aq)_	(8)	[54,55]

## Data Availability

Not applicable.

## Supplementary Materials

The following are available online at https://www.mdpi.com/article/10.3390/antiox10040605/s1. Figure S1: Fluorescence spectra of untreated water (blue line), and PTW (red line) 15 min after the discharge; Figure S2: Coomassie blue stained Saos2 cells, fixed after 72 h of incubation with PT-DMEMs at different H_2_O_2_–NO_2_^−^ ratio; Figure S3: Coomassie blue stained Eahy926 cells, fixed after 72 h of incubation in PT-DMEMs at different H_2_O_2_–NO_2_^−^ ratio; Figure S4: EPR spectra and fluorescence spectra of PT-DMEM with and without the serum; Figure S5: Coomassie blue stained Saos2 cells 48 h after incubation in 300 µM H_2_O_2_ solution in DMEM compared with the control.
