# Supplementary material for: Plasma Treated Water Solutions in Cancer Treatments: The Contrasting Role of RNS
Source: Antioxidants (Basel). 2021 Apr 14;10(4):605. doi: 10.3390/antiox10040605 (PMC8071004; doi:10.3390/antiox10040605)
Supplement: Supplementary file 1 [file antioxidants-10-00605-s001.pdf]

Plasma Treated Water Solutions in cancer treatments: the contrasting role of RNS

Eloisa Sardella[1,*], Valeria Veronico[2,] Roberto Gristina[1], Loris Grossi[3], Savino Cosmai[1], Marinella Striccoli[4], Maura Buttiglione[5], Francesco Fracassi[2,1], Pietro Favia[2,1]

1 CNR- Istituto di Nanotecnologia (CNR-NANOTEC) UoS Bari, c/o Dipartimento di Chimica, Università degli Studi di Bari Aldo Moro, via Orabona, 4, 70126 Bari, Italy
2 Dipartimento di Chimica, Università degli Studi di Bari Aldo Moro, via Orabona, 4, 70126 Bari, Italy,
3 Dipartimento di "Scienze per la Qualità della Vita" Università di Bologna, Corso d'Augusto, 237, I-47921 Rimini, Italy;
4 CNR-Istituto per i Processi Chimico-Fisici UoS Bari, via Orabona, 4, 70126 Bari, Italy
5 Dipartimento di Scienze Biomediche e Oncologia Umana, Scuola di Medicina, Università degli Studi di Bari Aldo Moro, 70124 Bari, Italy
* Correspondence: eloisa.sardella@cnr.it; Tel.: +39-080-544-2295 (E.S.)

Received: date; Accepted: date; Published: date

1. Alkali hydrolysis of 4-Amino-5-Methylamino-2',7'-Difluorofluorescein Diacetate (DAF-FM-DA)

DAF-FM was obtained by alkali hydrolysis of DAF-FM-diacetate: 1 µl of 5mM DAF-FM-DA solution in DMSO (anhydrous, ≥99.9% purity, Sigma Aldrich) was added with 5 µl of methanol (≥99.9%purity, Sigma Aldrich) and 10 µl of 0.1 M NaOH and incubated at room temperature for 1 h, in dark. To the mixture 20 µl of 1 M PBS, pH 7.0 ( ), and 10 µl of 0.1 M HCl were added to obtain DAF-FM, which was used to detect NO on the same day of the preparation: the probe was added to PTWSs at a concentration of 2.5 µM and the samples were incubated in dark at room temperature for 30 min.

2. Baseline subtraction and data processing in H_2O_2 analysis

H_2O_2 produced in PT-DMEM in different operating conditions was evaluated by means of a copper-phenantroline colorimetric assay, where the reduction of Cu(II) to Cu(I) ions forms an orange complex absorbing at 445 nm (Spectroquant ®, cat. N. 118789, Merck Millipore, Burlington, MA, USA). The absorption spectra of such stained PT-DMEMs were acquired by means of a UV-Vis spectrophotometer. In each acquisition, water containing the same amount of reagents added to the samples was used as control, while the spectrum of untreated DMEM was acquired with addition of the reagent for the detection of H_2O_2. Spectra acquired in this way were digitally processed with the Origin-Pro 8.0 software (Originlab Corporation); the baseline was subtracted first, then the spectrum of untreated DMEM. The absorbance value at 454 nm of such corrected spectra was then used to calculate the concentration of H_2O_2 in the samples by using the standard calibration equation obtained from previous experiments. In the case of NO_2^- this correction was not necessary, since no absorption was registered in the interested area of the spectrum on untreated DMEM after the addition of Griess reagents.

3. Fluorescence spectra of PTW

The NO signal in PTW observed in the EPR analysis with the $Fe^{++}(DETC)_2$ spin trap was also confirmed by the spectrofluorimetric detection method based on the NO sensor DAF-FM. The fluorescent intensity increases many-folds in PTW with respect to untreated water.

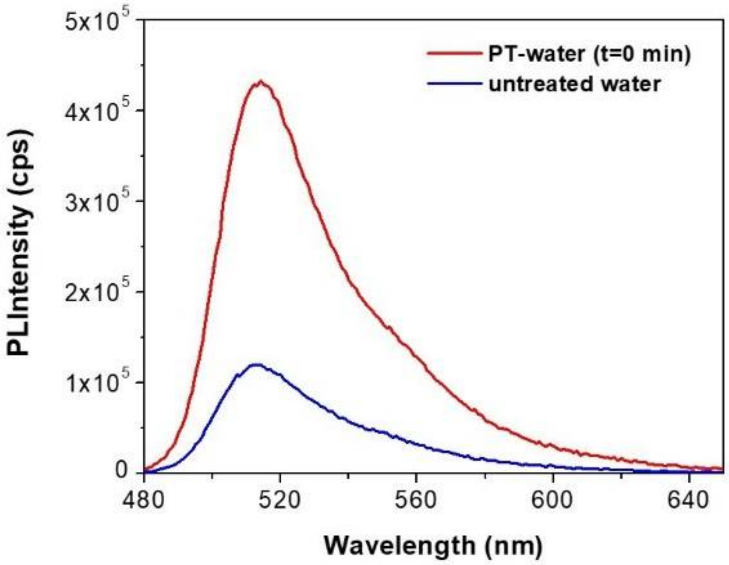

Figure S1. Fluorescence spectra of untreated water (blue line), and PTW (red line) 15 min after the discharge. PTW was stored for 15 min at room temperature and then mixed with the solution kits following the procedure reported in the experimental. Excitation wavelength 450 nm. Experimental plasma conditions: 15 min, 0.5 slm Air, 13kV, 6KHz, 50%DC, 100 ms period.

4. Cell morphology assay

In Fig. S2 and S3 below, significative images of Saos2 and EA.hy 926 cells are shown, fixed and Coomassie Blue stained after 72 h of incubation with PT-DMEMs at different H_2O_2/NO_2^- ratio. At least 7 images per type/ per time, were analysed to create the graphs of Fig. 4 of the main text.

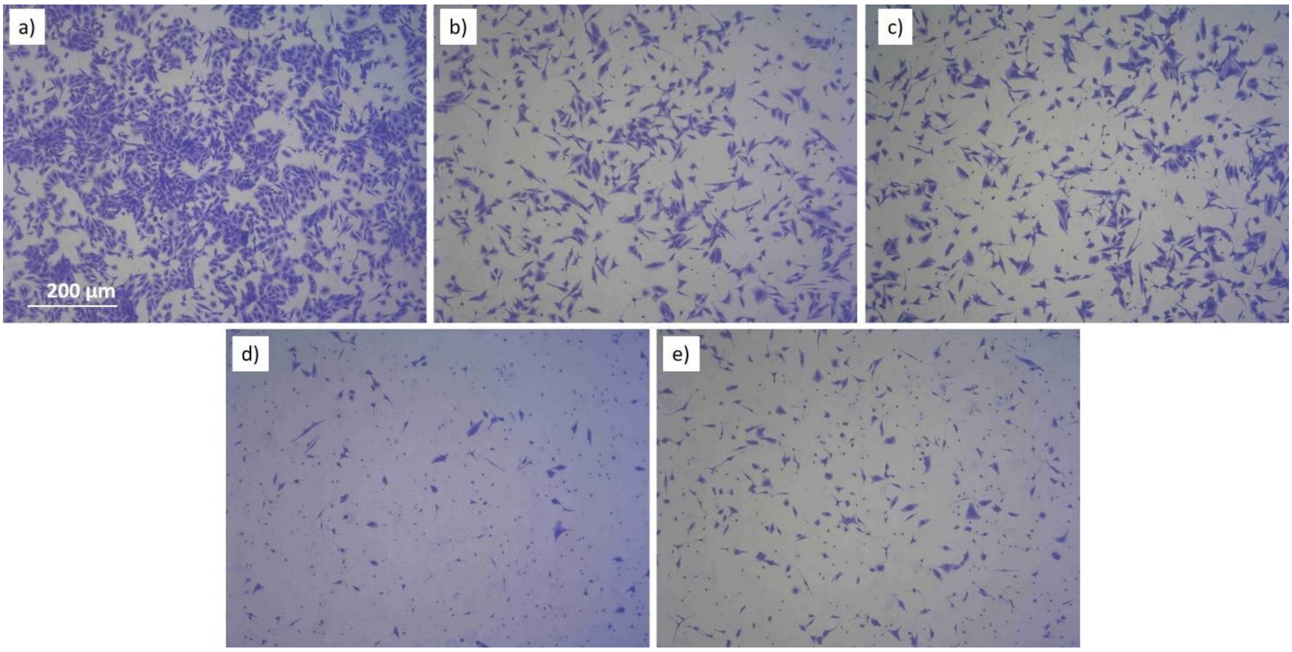

Figure S2. Coomassie blue stained Saos2 cells, fixed after 72h of incubation with PT-DMEMs with different H_2O_2/NO_2^- ratio. a) control cells; b)cells incubated with (−)H_2O_2(−)NO_2^-;c) cells incubated with (+)H_2O_2 d)

cells incubated with (++)H_2O_2; e) cells incubated with (+(+)H_2O_2(+)NO_2^- It's evident that a loss in cell clustering is present in all the Saos2 cells incubated with PTWSs.

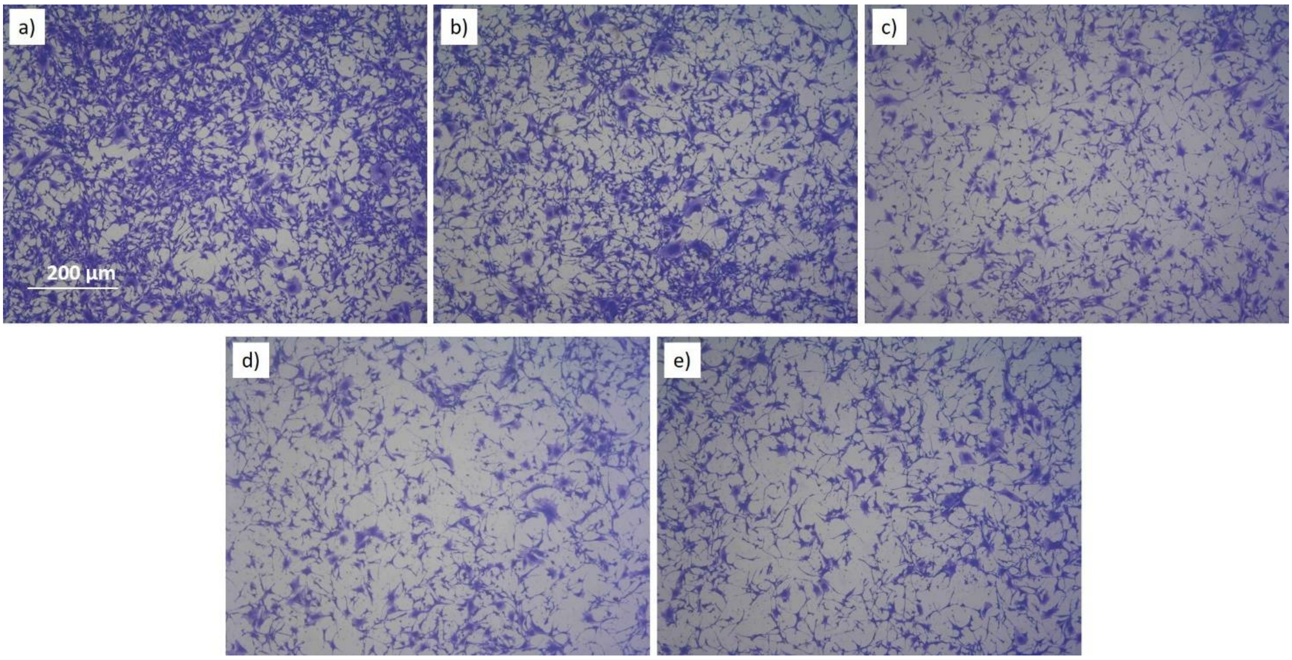

Figure S3. Coomassie blue stained Ea.hy 926 cells, fixed after 72h of incubation in PT-DMEMs with different H_2O_2/NO_2^- ratio. a) control cells; b)cells incubated with (−)H_2O_2(−)NO_2^-; c) cells incubated with (+)H_2O_2 d) cells incubated with (++)H_2O_2; e) cells incubated with (+(+)H_2O_2(+)NO_2^- Endothelial cells show only a change on cell density and not in morphology, differently from what has been observed for Saos2 cells,

5. EPR and fluorescence spectra of PT-DMEM with and without Fetal Bovine Serum

In order to assess whether the lack of the NO signal in PT-DMEM was caused by possible scavengers in the Fetal Bovine Serum (FBS) added to DMEM, EPR analysis and fluorescent assay with DAF-FM were carried out on the medium with 10% *v/v* FBS, a concentration used in this research, and on DMEM with no serum or other additives added. EPR and fluorescence results for both liquids are illustrated in Fig. S5. As it is shown, despite the absence of serum in the medium, no signal of NO could be found in serum-free PT-DMEM, neither with EPR (Fig. S5b) nor with the fluorescent assay (Fig. S5d). This results allow to exclude serum components as the cause for the lack of the NO signal in PT-DMEM. Probably NO is scavenged by DMEM original components, or it is not formed at neutral pH.

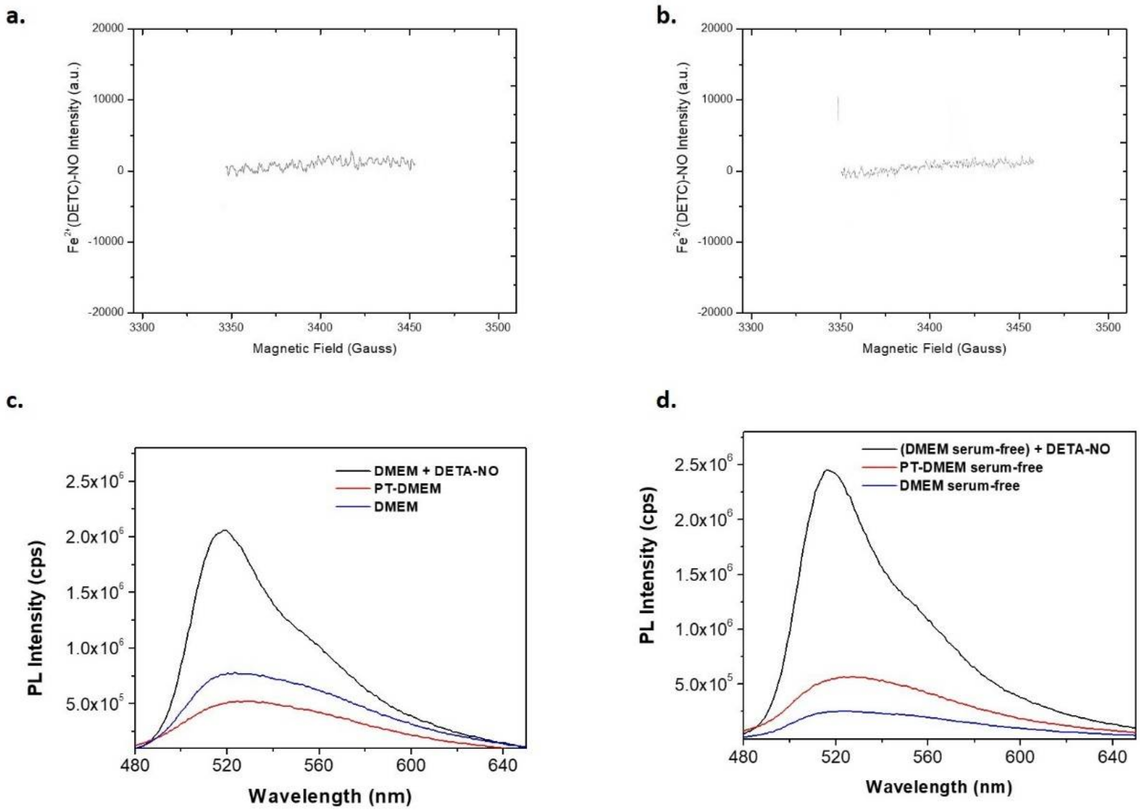

Figure S4. EPR spectra and fluorescence spectra of PT-DMEM with and without the serum. EPR spectra of: (a) PT-DMEM +FBS (10%*v*/*v*), (b) PT-DMEM –FBS; Fluorescence spectra of (c) PT-DMEM with added FBS (10%*v*/*v*), (d) serum-free PT-DMEM after the use of DAF-FM probe excited at 450nm. Experimental conditions: 15 min, 0.5 slm Air, 13 kV, 6 KHz, 50% DC, 100 ms period.

6. Saos2 cells exposure to 300 μM H_2O_2 solution of in DMEM

To investigate the lethal threshold of H_2O_2 concentration for Saos2 cells, solutions with different H_2O_2 concentrations in the medium were prepared from a commercial H_2O_2 water solution (cat. N. Sigma Aldrich, St. Louis, MO, USA) and then used to grow Saos2 cells in Petri dishes (57 mm dia) at a cell density of 10^5/dish. Approximately 20 h after the seeding, cells were incubated with 2 mL of the H_2O_2 solution (300 μM) for 2 h (37°C, 5% CO_2); after, the solution was replaced with DMEM and the culture was prolonged for 48 h, then cells were fixed and Coomassie Blue stained. As it is shown in Fig. S5, Saos2 cells, exposed to the 300 μM H_2O_2 solution in DMEM were very few, no clustered and with a spherical shape, clear evidences of apoptotic or pre-apoptotic conditions.

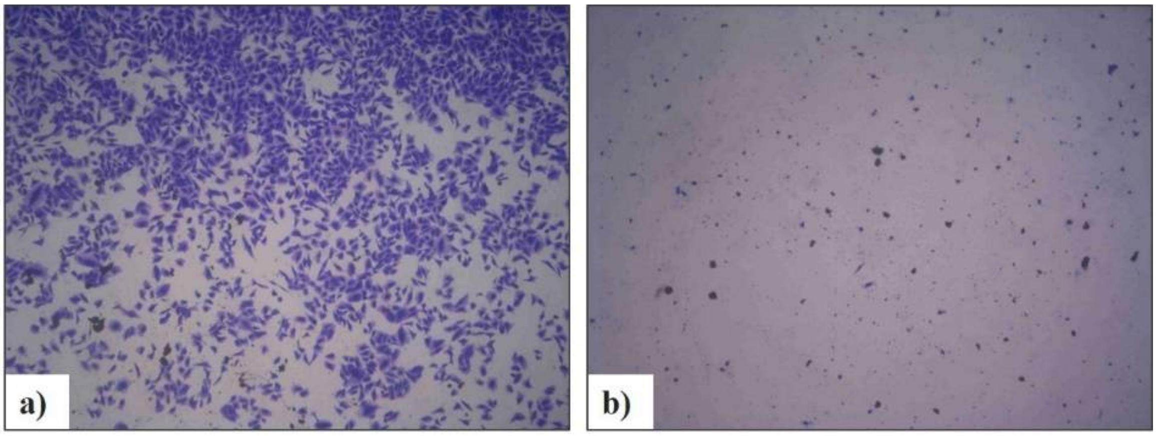

Figure S5. Coomassie blue stained Saos2 cells on the control (a) and (b) 48 h after incubation in 300 μM H_2O_2 solution in DMEM.
